# Efficacy of Antimicrobials Against Enveloped and Non-Enveloped Viruses on Porous Materials: A Review

**DOI:** 10.3390/microorganisms13122827

**Published:** 2025-12-12

**Authors:** Jinge Huang, Breanna Kimbrell, Runan Yan, Angela M. Fraser, Xiuping Jiang

**Affiliations:** 1 Department of Food, Nutrition, and Packaging Sciences, Clemson University, Clemson, SC 29634, USA; jingeh@g.clemson.edu (J.H.); afraser@clemson.edu (A.M.F.); 2 Department of Biological Sciences, Clemson University, Clemson, SC 29634, USA

**Keywords:** viruses, persistence, disinfection, porous material, environmental factors

## Abstract

Fomites are common vehicles for viral transmission. Most studies on virus disinfection have focused on non-porous, hard surfaces, with few investigating porous materials. This review addresses two research questions: (1) What affects viral viability on reusable porous materials? (2) Which antimicrobials effectively target viruses on these materials? Among existing studies, viral persistence on reusable porous surfaces was influenced by several factors, including viral envelope status, virus subtype, material type and structure, temperature, relative humidity, deposition method, and transmission medium. Disinfectants evaluated included ultraviolet irradiation, steam, chlorine, quaternary ammonium compounds, alcohols, glutaraldehyde, silver, and peroxide-based agents. Chlorine and steam were most effective; glutaraldehyde and peroxides showed limited action against non-enveloped viruses. Viral persistence and disinfection efficacy on reusable porous materials are influenced by multiple factors, highlighting the need for robust environmental management and infection control practices. Lack of standard tests and long-term disinfection effects on material integrity remain key challenges needing further study.

## 1. Introduction

Viruses are a common source of acute infections worldwide [1,2]. Many are transmitted person-to-person through direct or indirect contact. In direct contact transmission, pathogens are transmitted from an infected individual to a susceptible host, whereas indirect contact transmission occurs via a contaminated intermediary (e.g., hands or environmental surfaces). Indirect contact transmission can persist for days or even weeks, depending on the virus and environmental conditions [3,4,5,6]. Consequently, some viruses are difficult to control because of their ability to remain viable on surfaces and spread efficiently through fomite-to-person transmission [2,7,8].

In public spaces (e.g., restaurants and transportation systems), materials are constructed of both non-porous materials (e.g., stainless steel benches, glass, and plastics) and porous materials (e.g., brick, wood, and textiles) [9]. The diverse characteristics of these materials present efficacy challenges when treated with an antimicrobial [10,11,12]. In contrast to non-porous materials, porous materials tend to retain pathogens because their absorbent structure can trap fluids and small particles, shielding microorganisms from antimicrobial action [12,13,14]. The current understanding regarding the persistence of viruses on porous materials is limited, with only one systematic review focused on enteric viruses on soft materials [7]. Moreover, as some porous materials, including paper, are disposable, disinfection of viruses is typically needed on reusable materials. Hence, a comprehensive review to examine the factors influencing the persistence of animal and human viruses on reusable porous materials is needed.

The risk of spreading viruses can be significantly reduced by proper implementation of infection control practices, such as handwashing, use of personal protective equipment, environmental cleaning, and appropriate handling of textiles and laundry [15,16]. Environmental sanitation—practices aimed at reducing pathogen presence on surfaces—is essential for effective infection control [17]. Implementing these practices requires knowledge of virus persistence and the efficacy of antimicrobial agents [17]. In the U.S., the Environmental Protection Agency (EPA) oversees the regulation of chemical sanitizers and disinfectants applied to soft porous materials (Table 1), and enforces a standard efficacy testing requirement, which requires achieving a reduction of ≥3-log and ≥6-log of the target virus and bacteria, respectively [18]. It should be noted that the EPA does not set a specific standard for disinfecting viruses on porous materials; instead, it evaluates such cases individually. Moreover, disinfectant efficacy claims for soft, porous surfaces typically do not guarantee complete virus inactivation. Considering various studies that have explored virus disinfection on porous materials, there is a need for a comprehensive review that aims to summarize information about the effectiveness of various virus disinfection approaches on porous materials.

In brief, this review is guided by the following two research questions: (1) What factors affect the persistence of human and animal viruses on reusable porous materials? (2) How effective are current disinfection approaches in reducing viral load on reusable porous surfaces? Particularly, this literature review will identify the gaps that exist in the current literature regarding virus persistence and disinfection on reusable porous materials.

## 2. Persistence of Viruses on Porous Materials

**Figure 1:**
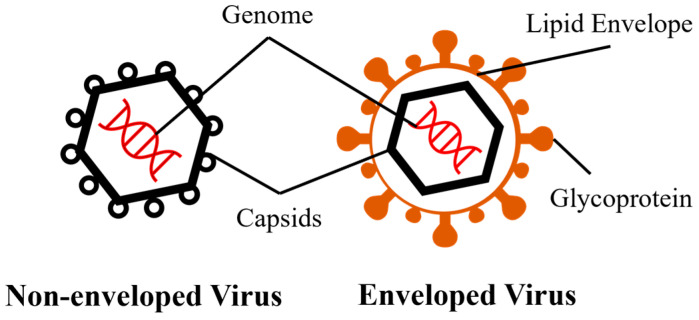
Basic structures of non-enveloped and enveloped viruses.

The presence of a viral envelope was significantly associated with virus persistence. Other factors, including material characteristics, temperature, relative humidity (RH), transmission medium, deposition method, and viral strain or subtype, also influence virus viability (Table 2). Generally, non-enveloped viruses persist longer on porous materials than do enveloped viruses due to the structural difference (Figure 1). Specifically, non-enveloped viruses can remain infectious for periods ranging from one day to up to 15 days at room temperature, while most enveloped viruses typically persist less than 1–2 days. This phenomenon could be attributed to the inherent susceptibility of the envelope, which is composed of a monolayer of phospholipids [28]. The viability of the virus can be compromised due to the impact of dehydration, surfactants, heat, and so on, on the phospholipid envelope [29,30,31]. One exception is enveloped influenza A virus H5N1, which has shown remarkable persistence and can remain infectious for up to 17 to 44.7 days on feathers at room temperature [32]. The increased viability was attributed to the presence of preen oil on feathers, which aggregated viruses and provided protective effects. However, the study did not specify the effect of two critical factors: relative humidity (RH) and the transmission medium of the virus. Additionally, the study lacked a comprehensive description of the potential protective mechanism provided by the feathers, hence impeding the ability to compare this finding with other studies on virus persistence.

### 2.1. The Effect of Material Characteristics

Characteristics of inanimate materials, also critical in virus persistence, are typically categorized as: the material and the structural construction [71]. While many studies have investigated the effect of materials on virus persistence [38,45,53,68], the impact of material construction, often recognized as material roughness, topography, or porosity, has remained understudied. Furthermore, materials can also be categorized based on their launderability. However, this classification is linked to disinfection procedures and may not be correlated to the persistence of viruses. Nevertheless, we still presented virus persistence data on launderable materials, because these data can potentially serve as a guide in the development of effective disinfection strategies.

Given that the presence of a viral envelope has a significant impact on virus persistence, the effects of material characteristics were examined separately for enveloped and non-enveloped viruses. Several studies reported persistence of non-enveloped viruses was greater on porous materials than on non-porous materials. For example, feline calicivirus (FCV), murine norovirus (MNV), and poliovirus (PV) persisted longer on porous materials, such as carpet, cotton, and wood, compared to non-porous materials, such as stainless steel [39,41]. However, non-enveloped bacteriophage MS2, a bacteriophage, was less persistent on polyester tablecloths than on plastics at room temperature [35].

Many enveloped viruses are less persistent on porous materials than on non-porous materials. For example, SARS-CoV and SARS-CoV-2 persist for shorter periods on cotton cloth and wood, compared to non-porous materials (e.g., stainless steel, glass and plastics) [60,61,63]. In contrast, influenza A virus and porcine epidemic diarrhea virus were more persistent on feathers, wood, and cloth than on stainless steel [45,59]. Material type can also significantly affect the virus persistence on porous materials. For example, avian metapneumovirus, cytomegalovirus, equine herpesvirus, and SARS-CoV were more persistent on cotton cloth than on wood, whereas vaccinia virus persisted longer on cotton than wool [69]. Avian metapneumovirus, Ebola virus, influenza A virus, and SARS-CoV were more persistent on more hydrophobic materials such as feathers, polypropylene gowns, and polyester fabrics [45,50,54]. Zuo et al. [54] investigated the effect of material hydrophobicity on the persistence of influenza A virus H9N9 and concluded that the hydrophobicity of the material significantly influenced virus persistence more than the types of specific materials used. One possible explanation is that the increased hydrophobicity of materials promotes virus aggregation, which provides protection to the enclosed viruses against environmental stressors [72]. In contrast, human coronavirus OC43, influenza A virus, and SARS-CoV-2 showed the opposite pattern in persistence [45,48,51,63,70], which the phenomenon is still under investigation.

The persistence of viruses is also significantly affected by the construction of materials. For example, PV was more persistent on wool blanket than on wool gabardine materials at room temperature in both 35% and 78% RH [42]. Furthermore, bacteriophage Phi6 persisted longer on looped carpet than on cut carpet [36]. These studies suggested that the construction of soft, porous materials could provide protection for viruses from desiccation. This protection might be attributed to mechanisms such as a decreased material area or the potential for viruses being absorbed into the porous texture of soft materials [36,73]. However, the material construction (looped or cut carpet) did not significantly affect the persistence of bacteriophage MS2 at room temperature in relative humidity (RH) between 30 and 40% [36], suggesting that highly hydrophobic bacteriophage MS2 could be less susceptible to desiccation on porous materials [71,74].

### 2.2. The Effect of Temperature and Relative Humidity

Apart from material characteristics, the persistence of viruses is notably affected by temperature and RH of the environment. The effect of temperature (4 °C vs. room temperature) was evaluated for 11 viruses, and all were found to persist longer at the lower temperature, regardless of whether they were enveloped (Table 2). At lower temperatures, the chemical and biological activities are decreased to maintain the structural integrity of viruses, hence protecting the viability of viruses [7]. This may also explain the seasonal trends in outbreaks associated with airborne, waterborne, and foodborne viruses. Though 4 °C or lower temperatures are typically used for preservation of most microorganisms, 20–25 °C is more commonly found in indoor environments and in public spaces due to indoor environmental standards and regulations [75]. Thus, studying the persistence of viruses at ambient temperatures is important to understand the transmission dynamics and develop preventive strategies.

RH also plays a key role in the occurrence of viral outbreaks [76,77]. In general, higher RH reduced the persistence of both non-enveloped and enveloped viruses, though exceptions were noted (Table 2). For example, poliovirus (PV) persisted longer at 35% RH than at 78% RH on wool, but the opposite trend was observed on cellulose filter membranes [37,42]. In contrast, the persistence of some viruses—including adenovirus, hepatitis A virus, PV, and rotavirus—was not significantly influenced by RH when tested on cotton [33,42]. Overall, our review indicates that most viruses tend to exhibit extended persistence times on porous materials under low RH conditions. At high RH, the increased water activity enhances chemical reactions, such as the Maillard reaction and oxidation, which contribute to the inactivation of viruses exposed to air [30]. The increased reactions are likely due to the increased rate of diffusion of reactants [30]. Additionally, viral envelopes, composed primarily of phospholipids, are more susceptible to oxidation than spike proteins [30]. Consequently, the influence of RH is greater for enveloped viruses than for some non-enveloped viruses, such as PV, rotavirus, hepatitis A virus, and adenovirus.

### 2.3. The Effect of Transmission Medium, Deposition Method, Strain Subtype and pH

Besides the presence of an envelope, virus persistence can also be affected by the contamination process. Several studies investigated the virus persistence under simulated contamination. For example, in some studies the authors used transmission media with a comparable composition to human body fluids (e.g., organic matter, fecal material, or artificial saliva) to suspend virus particles [46,69]. In addition, several studies used different inoculation techniques to mimic virus deposition process on inanimate materials, such as spiking, spraying, or the controlled release of virus-laden dust particles. The impact of transmission medium, deposition method, strain subtype, and pH has been studied only for a few viruses. The effects of transmission medium were evaluated on the persistence of five non-enveloped viruses, while bacteriophage Phi6 was the only enveloped virus studied in relation to the organic composition in transmission medium (Table 2). Savage et al. [34] revealed the different effects of transmission medium between two subtypes of avian reovirus. Specifically, 20% fecal matter did not affect the viability of avian reovirus R2 on cotton, whereas it provided protection for avian reovirus S1133. It was observed that the presence of fecal material reduced the viability of adenovirus and PV [33]. However, specific soil loads, such as tripart soil load and artificial saliva, decreased the reduction rates of bacteriophage Phi6 on wood from 1.98 to 0.08 and 1.30 log plaque forming unit (PFU)/h, respectively [46]. For bacteriophage Phi6, the protective effect conferred by organic matter may act as antioxidants or enhance viscosity, hence impeding direct interaction between viruses and the atmospheric oxygen [30,78,79]. However, organic matters such as fecal constituents may compete with the virus for adsorption sites, resulting in a shorter persistence period for AV and PV on cotton [33]. Moreover, recovery efficiency from porous material, which was rarely reported in persistence studies, could also be reduced by the presence of organic matter [80]. This phenomenon could underestimate the persistence of viruses on porous materials. Consistent with a previous review, the effect of organic matter on the persistence of viruses among different materials was not conclusive [7].

The deposition of virus particles is another factor in determining the persistence of viruses on porous materials. The deposition method had an impact on the persistence of influenza A virus, which showed longer viability when the 20 µL virus was spiked onto materials in one drop compared to being dispersed by aerosol [54]. There is a higher persistence rate when viruses are inoculated in liquid droplets compared to aerosols. This is primarily attributed to the smaller area occupied by liquid droplets on the surface, which makes them less susceptible to desiccation, thereby enhancing viral stability [80,81]. Additionally, due to the hydrophobic nature of the virus envelope or capsid, the larger areas of the air-water interface of aerosols may facilitate the gathering of virus particles on the interface, leading to an increased exposure to air and susceptibility to oxidation-induced damage [82].

RH may interact with the deposition method and result in different virus persistence. In 35% RH, Vaccinia virus exhibited longer persistence through virus-containing dust contact, whereas in 78% RH, the persistence was longer in droplets [69]. The effect of pH was only investigated by one study, which reported increased sensitivity to lower pH levels (<3) on cotton sheet and carpet (material unknown) for FCV [38]. Furthermore, the persistence is also different for different strains, as influenza A virus H9N2 was found to be more persistent than influenza A virus H6N2 on pine wood [55].

## 3. Disinfection of Viruses on Porous Materials

The disinfection efficacy has been studied for all non-enveloped viruses for which persistence has been reported on porous materials, except for avian reovirus (Table 3). Disinfection can be accomplished by either a laundry procedure or a non-laundry procedure, depending on the type of porous material. Specifically, the disinfection of adenovirus, bacteriophage MS2, hepatitis A virus, MNV, and PV was investigated by laundering cotton fabrics, while bacteriophage MS2, coxsackievirus, echovirus, FCV, foot-and-mouth disease virus and poliovirus were studied on non-launderable materials, including porous unglazed red clay, carpet, wood and cellulose membrane (Table 3). Among enveloped viruses, only bacteriophage Phi6, Ebola virus, and SARS-CoV-2 have been investigated regarding their persistence and disinfection on porous materials, but Vaccinia virus was only investigated for its persistence (Table 3). In addition, African swine fever virus and murine hepatitis virus have primarily been studied in terms of disinfection on concrete and bus seat fabric, respectively, but with limited data on their persistence.

Disinfection can be achieved by chemical disinfectant agents and physical treatments. EPA primarily focuses on regulating chemical disinfectants and enforces strict requirements for standardized efficacy testing [19]. Additionally, EPA or ASTM provides comprehensive guidelines for standardized testing methods tailored to specific disinfection procedures and the materials they target (Table 1). For porous materials, disinfection procedures can be classified into two major categories: laundry and non-launderable material disinfectants, according to the guidelines. It is important to acknowledge that many porous materials are not suitable for laundering. Additionally, disinfectants used in both laundry and non-laundry processes are subject to different standards and testing methods according to the EPA and ASTM [19]. Consequently, this review presented a clear distinction between disinfectants suitable for launderable materials and those designed for non-launderable materials, with our focus on the latter.

### 3.1. Launderable Materials

In general, laundry disinfection is composed of two steps: a water rinse cycle and a hot air-drying cycle. EPA stipulated that disinfectants intended for laundry use must successfully undergo a standard suspension test, i.e., AOAC use-dilution methods (Table 1). This is due to the ability of water to remove viruses from the fabric and hold them in suspension during the entire laundry process. Consequently, a significant level of virus inactivation occurs through contact with disinfectants in the suspension rather than on the fabrics [83,102]. The removal of viruses by water and detergents from fabrics is a crucial step in the laundry process. During the water rinse step, the efficacy of virus disinfection can be affected by the water temperature and the addition of disinfectant. Hot water wash (54–60 °C) can more effectively inactivate PV on cotton, wool, and nylon than warm (38–43 °C) and cold (21–27 °C) water [92].

Surfactants in detergents can cause damage to viral envelopes composed of phospholipids [103]. Furthermore, it was also reported that the addition of sodium hypochlorite (NaClO) in the wash cycle reduced the adenovirus, hepatitis A virus, and rotaviruses by >4 logs after the final rinse [102]. SARS-CoV-2 can also be effectively reduced by adding 0.07% NaClO during the laundry water rinse step [98]. However, the reduction in SARS-CoV-2 by 70% alcohol and Lysoform^®^ (unknown product) was less during laundry [98]. NaClO (commonly known as bleach) was the only disinfectant extensively explored for laundry disinfection. While high water temperatures can contribute to virus inactivation during the laundry process, the addition of bleach can significantly enhance the inactivation of viruses [93]. However, sodium hypochlorite is a strong oxidizer that has the potential to damage fabrics and bleach clothing [104]. Therefore, there is a need for the development of alternative disinfectants to bleach to achieve effective virus inactivation and mitigate clothing damage in the laundry process.

Interestingly, the hot air-drying cycle for 28 min after a detergent wash cycle only reduced a maximum of 0.19 log PFU per 58 cm^2^ area for these three viruses, which is considered ineffective according to the EPA regulations (≥3 logs) [83].

### 3.2. Non-Launderable Materials

Chemical disinfectants are essential tools for effectively disinfecting non-launderable materials. The EPA oversees the regulation of disinfectants in the US, which includes a variety of active ingredients and formulations specifically designed for non-porous materials. However, only a limited subset of the available products has been tested on porous materials [87,88]. According to the EPA, there is a lack of a standardized testing method for non-launderable soft porous materials [19]. The lack of a standard testing method can be linked to challenges in recovering viruses from non-launderable porous materials [39,91]. Moreover, the efficacy of disinfectants against viruses can be significantly influenced by the characteristics of porous materials [87,88]. This further complicates the establishment of a universal standard testing method that accurately measures the efficacy of disinfectants on specific types of porous materials. In our review, an evaluation was conducted on various active ingredients, including chlorine, quaternary ammonium chemicals (QACs), alcohols, glutaraldehyde, silver, and peroxides, to assess their effectiveness against some viruses on non-launderable porous materials, with each having limitations.

The effectiveness of NaClO or other chlorine-based disinfectants was extensively reported against both non-enveloped and enveloped viruses on non-launderable materials. For example, 1.4–1.5% NaClO was able to effectively reduce >3 logs of Ebola virus in 10 min when applied to pilot seat-belt strapping [95]. A NaClO solution at 0.5% or 5000 ppm, a commonly used concentration, effectively reduced 3.92 logs of bacteriophage MS2 on wood and >3 logs of FCV and MNV on cotton fabrics in 5 min [89]. Furthermore, the chlorine solution with 0.1–0.5% NaClO inactivated >4 logs of SARS-CoV-2 in 0.5 min on wood [84]. Similarly, a solution containing 0.5% NaClO resulted in a reduction in bacteriophage Phi6 by 2.98 and 6.83 logs on wood and concrete materials, respectively, within 1 min at a temperature of 25 °C and RH of 23% [94]. At increased RH (85%), the reduction in bacteriophage Phi6 was similar on wood but decreased to 4.32 logs on concrete materials within 1 min [94]. A lower concentration of NaClO solution at 1076 ppm was also effective against bacteriophage MS2 on the polyester carpet [36]. At 500 ppm, NaClO was effective against Echovirus 25 on cellulose membrane, but not effective against Echovirus 6 [37]. For murine hepatitis virus, applying detergent or chlorine-based disinfectants on contaminated seat fabric followed by immediate wiping was found ineffective [96]. Other than NaClO, PurTabs^®^, which can also produce hypochlorous acid as the active ingredient, resulted in a reduction of over 3 logs of bacteriophage Phi6 within 1 min when applied to polyester carpet [36]. In summary, chlorine-based disinfectants are highly effective against Echovirus, FCV, MNV, PV, bacteriophage Phi6 and murine hepatitis virus, but they can damage materials when used at high concentrations (e.g., 5500 ppm) or after prolonged use due to the strong oxidizing properties [104,105].

Besides chlorine-based disinfectants, 2.6% glutaraldehyde has been proven effective (>3 log reduction) in 1 min against FCV on carpets made from olefin, polyester, and nylon, as well as fabrics made from cotton, polyester, and cotton mix [87]. Additionally, silver dihydrogen citrate (0.003% silver ion) was also effective against FCV with a 3.62 log-reduction on nylon carpets in 60 min [88]. However, it demonstrated limited efficacy (<3 log reduction) on blended carpet made from unspecified materials after 10 min of contact time [87]. Moreover, 0.05% glutaraldehyde with a 60 min contact time did not exhibit strong efficacy against coxsackievirus, echoviruses, and PV on cellulose membranes [37]. The efficacy of glutaraldehyde and silver in virus disinfection on specific materials such as cellulose membrane, nylon carpet, and olefin carpet has been demonstrated [37,87,88]. Nonetheless, exposure to glutaraldehyde can potentially pose human health risks [106]. Additionally, the use of silver dihydrogen citrate has been found to cause the formation of a sticky film on carpets [88].

Peroxide-based disinfectants have shown effectiveness against both non-enveloped and enveloped viruses, demonstrating their potential as broad-spectrum antiviral agents. Specifically, disinfectants utilizing H_2_O_2_ as the active ingredient were effective against FCV on cotton fabric in 5 min and murine hepatitis virus on seat fabric in 30 s [89,97]. The effectiveness of ozone at concentrations of 20–25 ppm for 20 min was shown against FCV on fabric, cotton, and carpet in office environments [95,97]. Peroxides, including peracetic acid and hydrogen peroxide (H_2_O_2_), can denature viral capsid proteins, but only limited data on porous materials are available [89,90,97,107].

QACs and alcohols are less likely to cause material damage; however, they demonstrated weak activity against non-enveloped viruses [87,108]. For example, chlorophenol/phenylphenol-based, quaternary ammonium compound (QAC)-based, and alcohol-based disinfectants were not effective against FCV on either fabric or carpets [87]. Despite the limited efficacy against non-enveloped viruses, two QAC-based disinfectants, Ardrox 6092 and Desintex, achieved >3-log reduction in efficacy against Ebola virus in 10 min on pilot seat-belt strapping, while another QAC-based disinfectant presented efficacy against murine hepatitis virus [95,97]. Interestingly, the efficacy of disinfection may also be affected by the method used to dispense the disinfectant, as one study reported that the use of an electrostatic sprayer decreased the disinfection efficacy of Vital Oxide (0.2% chlorine dioxide) against murine hepatitis virus compared to a trigger-pull sprayer [97]. The observed reduction in efficacy in electrostatic spray was likely attributed to gaps among evenly distributed disinfectant droplets and a reduced amount of free radicals produced by active ingredients between the charged droplets [97]. Other than the aforementioned chemical disinfectants, the disinfectant Virkon, which contains potassium peroxymonosulfate as an active ingredient, was found to be effective against African swine fever virus when used at concentrations greater than 2%, resulting in a reduction of over 2.2 logs on concrete materials after a 5 min contact time [91].

In addition to chemical disinfectants, the CDC also recommends using steam cleaning to address carpet contamination after a human norovirus outbreak [109]. However, the CDC has not published any specific protocols in compliance with this guidance. One report has provided evidence that steam cleaning was able to reduce bacteriophage MS2 by 6 logs on unglazed clay in 5 s and polyester carpet with a 60 s treatment, and the sensitivity to heat and humidity of bacteriophage MS2 can be further increased by using cell culture medium as the transmission medium, likely due to medium-induced alterations of its surface hydrophobicity [74,85]. Additionally, it has been found to be effective against FCV on nylon and wool carpets in 90 s, and bacteriophage Phi6 on polyester carpet in 60 s [36,88]. These results are probably attributed to the rapid denaturation of viral proteins in the outer structures due to heat [36,85,88]. However, the potential effect of various external factors, such as material construction, steam temperature, and heat distribution on materials, on the effectiveness of steam vapor has not been comprehensively examined.

Ultraviolet (UV) light is another physical disinfection method that can be performed without direct contact with fomites. UV radiation at 100 mJ/cm^2^ for 70 s reduced bacteriophage MS2 by 1.27–1.58 logs on the front sections and −0.07 to 1.36 logs on the side sections of a cotton T-shirt [85]. Furthermore, UV-C treatment (396 mJ/cm^2^) showed efficacy against SARS-CoV-2 only on fabrics of bus seats and clothing in 28 min and 22 s, respectively [99]. UV-C has been found effective in eliminating bacteria on hard porous materials [110], though one study reported its failure to disinfect bacteriophage MS2 on T-shirts [86]. This may be attributed to the inherent resistance of viruses to UV-C and the challenges associated with the effective penetration of UV-C into fabric materials [86].

The combination of physical and chemical disinfectants was only investigated in one study, which demonstrated that heat treatment combined with ozone (800 ppm) for a duration of 10 min was effective against SARS-CoV-2 on cotton fabric [100].

## 4. Conclusions

Our review indicates that virus persistence on porous materials is strongly influenced by virus type, material composition and construction, temperature, relative humidity, and deposition method. Enveloped viruses generally exhibit lower persistence than non-enveloped viruses, though exceptions exist. Lower temperatures, lower relative humidity, and liquid droplet deposition tend to promote longer persistence. The complex interplay among these factors underscores the need for careful monitoring of environmental and material parameters, as well as standardized testing when evaluating virus persistence. Among the various disinfection methods reviewed, chlorine-based agents and steam were identified as the most effective against a wide range of viruses on porous materials. In contrast, alternatives such as glutaraldehyde and peroxide-based disinfectants showed limited or inconsistent efficacy, particularly against non-enveloped viruses. However, standardized protocols for assessing disinfectant performance on non-launderable porous materials are lacking, and data on the long-term effects of repeated disinfection on material integrity remain scarce. Further research is needed to validate virus recovery methods and to confirm the effectiveness of disinfectants across different porous materials.

## Figures and Tables

**Table 1:** Major testing standards recommended by the U.S. EPA for registration of sanitizers and disinfectants for use on porous materials [19].

Treatment	Antimicrobials	Testing Methods	Evaluation of the Successful Efficacy ^1^
Launderable material:			
Pre-soak treatments	limited and broad-spectrum disinfectant	AOAC Use-Dilution Methods (AOAC 955.14; 955.15; 964.02) [20,21,22]	6 log reduction in *S. enterica* ^2^ and *S. aureus* ≤ 10 min
	healthcare disinfectants	AOAC Use-Dilution Methods (AOAC 955.14; 955.15; 964.02) [20,21,22]	6 log reduction in *Pseudomonas aeruginosa* and *S. aureus* in ≤10 min
	Sanitizers	ASTM E1153 [23]	3 log reduction in *S. aureus* and *K. pneumoniae* in ≤5 min
Laundry additives	Disinfectants	ASTM E2274 or E2406 [24,25]	4 log reduction in *S. aureus* and *K. pneumoniae* in a wash cycle, in addition to *P. aeruginosa* for healthcare facilities
	Sanitizers	ASTM E2274 or E2406 [24,25]	3 log reduction in *S. aureus* and *K. pneumoniae* in a wash cycle
	Self-sanitizing additives	AATCC Test Method 100 or ASTM E2149 [26,27]	3 log reduction in *S. aureus* and *K. pneumoniae* in no more than 24 h intervals on a case-by-case basis
Non-launderable material:			
Carpet sanitizers		Not available ^3^	3 log reduction in *S. aureus* and *E. aerogenes*, in addition to *P. aeruginosa* for healthcare facilities
Mattress, pillows and upholstered furniture treatments		Not available	Efficacy is on a case-by-case basis. Tested microorganisms are *B. subtilis* and *C. sporogenes* for claims of sterilization, *S. aureus* and *S. enterica* for claims of broad-spectrum disinfection, in addition to *P. aeruginosa* for healthcare facilities, *S. aureus* and *K. pneumoniae* (or *E. aerogenes*) for claims of sanitization
Material sanitizers for fabric and textiles		ASTM E1153 [23]	3 log reduction in *S. aureus* and *K. pneumoniae* (or *E. aerogenes*)

^1^ U.S. EPA does not provide a specific standard for disinfection of viruses on porous materials but evaluates it on a case-by-case basis. ^2^ Particular microbial strains are required as follows: *Salmonella enterica* (ATCC 10708), *Staphylococcus aureus* (ATCC 6538), *Pseudomonas aeruginosa* (ATCC 15442), *Klebsiella pneumoniae* (ATCC 4352), *Enterobacter aerogenes* (ATCC 13048), *Bacillus subtilis* (ATCC 19659), and *Clostridium sporogenes* (ATCC 3584). ^3^ U.S. EPA evaluates the products on a case-by-case basis when no standard testing method is applicable.

**Table 2:** Persistence of viruses on porous materials.

Virus	Material	Key Findings on Virus Persistence ^1^	Refs.
**Non-enveloped virus:**			
Adenovirus	Cotton cloth	• High T < Low T • RH: no effect • TM: Fecal materials reduce persistence on cotton • Median titer reduction at 3.2–3.3 logs after drying for 3 to 5 h	[33]
Avian reovirus	Cotton, wood	• Subtype: R2 < S1133 • MC and TM: Fecal materials no effect R2 on cotton; R2 persisted longer on wood with fecal materials; S1133 persisted longer with fecal materials on cotton and wood	[34]
Bacteriophage MS2	Polyester tablecloth, polyester carpet	• MC: tablecloth (<14 d) < plastics (<23 d); construction of carpet (cut or looped) no effect • Decay rate on carpets = −(0.09–0.20) h^−1^	[35,36]
Echovirus	Cellulose membrane	• High T < Low T • Low RH < high RH • Persisted for 2–7 d	[37]
Feline calicivirus	Cotton fabric, unknown carpet, wool/nylon carpet, formica	• Low pH (<3) < High pH (>3) • High RH (70%) < Low RH (30%) • Formica ≈ vinyl carpet < unknown carpet ≈ cotton fabrics • Persisted for 1–15 d	[38,39]
Hepatitis A virus	Cotton cloth	• High T < Low T • RH: no effect • Fecal materials: no effect • Median reduction at 0.8–1.6 logs after drying for 3 to 5 h	[33]
Murine norovirus	Wool/nylon carpet, wood	• High RH (70%) < Low RH (30%) • Stainless steel < wood • Persisted for 3–15 d	[39,40]
Poliovirus	Cotton cloth, wool, cellulose membrane	• High T < Low T • Cotton: RH does not affect cotton; Wool: High RH (78%) < Low RH (35%); Cellulose: Low RH (20%) < High RH (>85%) • MC: More persistence on wool blanket > wool garments; Cotton > plastics and stainless steel • TM: Fecal materials reduce persistence on cotton	[33,37,41,42]
Rotavirus	Cotton/polyester cloth	• High T < Low T • RH: no effect • TM: Fecal materials no effect	[33,43,44]
**Enveloped Virus:**			
Avian metapneumovirus	Wood, cotton and polyester fabric, feather	• MC: Feathers (6 d) > other porous materials (≤24 h)	[45]
Bacteriophage Phi6	Polyester fabric with and without zinc pyrithione, wood, polyester carpet	• High T < Low T • High RH < Low RH • MC: looped > cut carpet; Zinc pyrithione fabric coatings no effect • TM: tripart soil load > artificial > PBS	[36,46,47]
Cytomegalovirus	Cotton blanket, sanded pine plywood and cotton cloth	• MC: Cotton cloth > pine wood • At least 1 h on a cotton blanket at 25–27 °C	[48,49]
Ebola virus	Cotton gown	• MC: respiratory mask > cotton gown	[50]
Equine herpesvirus type-1	Pinewood shavings, polyester-cotton fabric	• High T < Low T • MC: polystyrene-cotton fabric > wood shavings	[51]
Human coronavirus OC43	Polyester, wool and cotton	• Persistence: Wool > polyester > cotton	[52]
Influenza A virus	Silver containing fabric, soft toy, wood, cotton cloth, microfiber, pinewood, duck feather (preen oil removed), polypropylene, polyester, polyamide, and polyester fabric	• High T < Low T • High RH < Low RH • MC: duck feather > wood > cotton cloth > stainless-steel > pine wood; Hydrophobic materials (polypropylene, polyester) > hydrophilic materials (polyamide); Microfiber > cotton • Deposition method: liquid inoculum > aerosol • Subtype: H1N1 ≈ H9N9 < H9N2 < H13N7 < H6N2 < H5N1	[32,45,53,54,55,56,57,58]
Porcine epidemic diarrhea virus	Unknown cloth	• High T < Low T • MC: Cloth > metal and nitrile gloves	[59]
Respiratory syncytial virus	Cloth gown (cotton/polyester)	• MC: Cotton/polyester cloth < countertop	[60]
SARS-CoV	Wood board, cloth, polypropylene gown and cotton gown	• MC: Glass > Cloth > wood board; Polypropylene gown (2 d) > cotton gown (24 h)	[61,62]
SARS-CoV-2	Cotton cloth, treated wood, gym pit foam, nylon and PET carpet	• High T < Low T • MC: Gym pit foam < cotton cloth < wood < non-porous; Nylon carpet < PET carpet	[63,64,65,66,67]
Vaccinia virus	Cotton, wool, industrial carpet	• High T < Low T • High RH < Low RH • MC: Cotton > wool • Deposition method: At low RH (1–10%), virus-containing dust > droplets; At high RH (89–100%), less persistent in virus-containing dust < droplets	[68,69,70]

^1^ The effect factors studied were identified as temperature (T), relative humidity (RH), material characteristics (MC) and transmission medium (TM).

**Table 3:** Disinfection of viruses on porous materials.

Virus	Materials	Disinfectants	Key Findings on Disinfection Efficacy	Refs.
Non-enveloped virus:				
Adenovirus	Cotton clothes	Laundry: Drying cycle and NaClO	• Washing with detergent not effective • NaClO (180 ppm) reduced >4 logs after the final rinse • Drying for 28 min not effective	[83]
Bacteriophage MS2	Porous unglazed red clay coupon, cotton T-shirt, polyester carpet, wood	Non-laundry: Steam, UV-C, chlorine-based disinfectant	• Steam vapor effective on unglazed clay and polyester carpet, affected by cell culture medium • UV-C (~100 mJ/cm^2^) with weak efficacy on T-shirt (≤1.58 logs for 70 s) • Chlorine (1076 ppm) effective on polyester carpet • NaClO (5000 ppm) effective on wood (3.92 logs after 5 min)	[36,84,85,86]
Coxsackievirus	Cellulose membrane	Non-laundry: Glutaraldehyde, chlorine	• Chlorine (500 ppm) with weak efficacy in 10 min • Glutaraldehyde (500 ppm) not effective in 10 min	[37]
Echoviruses	Cellulose membrane	Non-laundry: Glutaraldehyde, chlorine	• Chlorine (500 ppm) not effective in 10 min against Echovirus 6, but effective against Echovirus 25 • Glutaraldehyde (500 ppm) not effective in 10 min	[37]
Feline calicivirus	Nylon, wool, and olefin polyester carpets, cotton fabric, cotton, polyester and cotton blended cloth	Non-laundry: Silver dihydrogen citrate, steam vapor, hydrogen peroxide-, chlorine-based, glutaraldehyde-based, chlorophenol/phenylphenol-based, QAC-based, and alcohol-based disinfectants, ozone	• Steam vapor effective (≥3.68 logs) in 90 s on wool and nylon carpets • Silver dihydrogen citrate (30 ppm) not effective on the wool carpet, but effective on the nylon carpet • Both H_2_O_2_- and chlorine-based disinfectants effective on cotton fabric • Glutaraldehyde (26,000 ppm) effective (>3 logs) in 10 min on olefin, polyester and nylon carpets, cotton, polyester and cotton blended fabrics, but not effective on blended carpet • Chlorophenol/phenylphenol-based, QAC-based, alcohol-based disinfectants not effective • Ozone (20–25 ppm) effective on fabric, cotton, and carpet in an office	[87,88,89,90]
Foot-and-mouth disease virus	Concrete	Non-laundry: Virkon (potassium peroxymonosulfate)	• >Virkon (1%) effective in 10 min on concrete	[91]
Hepatitis A	Cotton clothes	Laundry: Drying cycle and NaClO	• Washing with detergent not effective • NaClO (180 ppm) reduced >4 logs after the final rinse • Drying for 28 min not effective	[83]
Murine norovirus	Cotton fabric	Non-laundry: Hydrogen peroxide- and chlorine-based disinfectants	• H_2_O_2_-based disinfectants not effective • Chlorine-based disinfectants effective	[89]
Poliovirus	Cotton, wool, and nylon clothes in laundry studies; Cellulose membrane	Laundry: Detergent, washing time, and water temperature Non-laundry: Glutaraldehyde, NaClO	• Detergent effective • Hot water (54–60 °C) more effective than cold (21–27 °C) and warm (38–43 °C) water • NaClO (500 ppm) more effective than 0.05% glutaraldehyde on cellulose membrane	[37,92,93]
Rotavirus	Cotton clothes	Laundry: Detergent, washing time, and water temperature	• Washing with detergent not effective • NaClO (180 ppm) reduced >4 logs after the final rinse • Drying for 28 min not very effective	[83]
Enveloped virus:				
African swine fever virus	Concrete	Non-laundry: Virkon (Potassium peroxymonosulfate)	• >2 and 5% Virkon reduced >2.2 logs	[91]
Bacteriophage Phi6	wood, concrete, polyester carpet	Non-laundry: NaClO (0.5%), steam, PurTabs^®^ (sodium troclosene and hypochlorous acid, 1076 ppm free chlorine)	• At 25 °C, 23% RH, 0.5% NaClO reduced 2.98 and 6.83 logs on wood, concrete in 1 min, respectively • At 25 °C, 85% RH, 0.5% NaClO reduced 2.93 and 4.23 on wood and concrete in 1 min, respectively • Steam vapor and PurTabs^®^ reduced >3 logs in 1 min on polyester carpet	[36,84,94]
Ebola virus	Pilot seat-belt strapping	Non-laundry: QAC-based disinfectants and sodium hypochlorite	• Ardrox 6092(QAC-based), Desintex(QAC-based) and 1.4–1.5% sodium hypochlorite reduced >3 logs in 10 min on pilot seat-belt strapping	[95]
Murine hepatitis virus	Bus seat fabric	Non-laundry: Detergent, QAC-, oxide-, chlorine-based disinfectants	• Detergent nor chlorine-based disinfectants ineffective by wiping • QAC- and oxide-based disinfectants effective • Electrostatic sprayer decreased efficacy compared to trigger-pull sprayer	[96,97]
SARS-CoV-2	Tricoline fabric, wood, fabrics from bus seat, car, hospital bed linen, hospital clothing, cotton fabric and strap flap	Laundry: Detergent, formaldehyde-based disinfectant, sodium hypochlorite, 70% alcohol Non-Laundry: UV-C, ozone, heat (40 °C)	• Only sodium hypochlorite (0.07%) effective during the wash, not 70% alcohol and Lysoform^®^ • 0.5% NaClO effective on wood after 0.5 min • UV-C (396 mJ/cm^2^) effective only on fabrics from bus seat and clothing, but not on fabrics from car • UV-C (460 mJ/cm^2^) effective on cotton fabric, but not on strap flap • Heat + ozone (800 ppm) for 10 min effective on cotton fabric	[84,98,99,100,101]

## Data Availability

The original contributions presented in this study are included in the article. Further inquiries can be directed to the corresponding author.

## Abbreviations

The following abbreviations are used in this manuscript:

EPA	United States Environmental Protection Agency
T	Temperature
RH	Relative humidity
MC	Material characteristics
TM	Transmission media
FCV	Feline calicivirus
MNV	Murine norovirus
PV	Poliovirus
SARS-CoV	Severe acute respiratory syndrome coronavirus
NaClO	Sodium hypochlorite
QAC	Quaternary ammonium compound
UV	Ultraviolet
